# The late atlantoaxial subluxation in a patient with juvenile idiopathic arthritis

**DOI:** 10.1186/1546-0096-12-S1-P159

**Published:** 2014-09-17

**Authors:** Ana-Maria-Mihaela Ramazan, Adriana Apostol

**Affiliations:** 1 Rheumatology, Emergency County Clinical Hospital, Constanta, Romania; 2 Onc-Hematology, Emergency County Clinical Hospital, Constanta, Romania

## Introduction

Atlantoaxial instability has been described as a manifestation of ankylosing spondylitis (juvenile and adult onset), reactive arthritis, juvenile idiopathic arthritis, and rheumatoid arthritis; however, it has rarely been reported as an early manifestation of these disorders (1). Instability of the atlantoaxial joint may lead to impingement on the cord and brainstem. There may also be cephalad encroachment of the odontoid into the foramen magnum. Locke and colleagues studied the atlanto-odontoid distance in 200 normal children aged 3 to 15 years, but the age and sex were not significant factors (2-4).

## Objectives

To increase awareness of the condition in the hope that earlier recognition of this disease may prevent further serious injury

## Methods

We report the case of a 52-year-old woman who was diagnosed with JIA due to juvenile onset (at 16 years old), polyarthritis and a positive rheumatoid factor; the disease was persistent as active disease in adulthood.

## Results

Our patient experienced persistent and worsening occipitocervical pain and signs of myelopathy after 36 years of disease and after one months of tumor necrosis factor α blockade. The atlantoaxial instability was appeared sudden in the night during sleep; she had awaked with dispneea and she had falled on his bed. She was intubated and the diagnosis was estabileshed after tomography, in which we had noticed along cervical spine abnormalities like superior subluxation, odontoid fracture with cord compression, bone erosion and pannus formation. She was treated surgically with a C1-2 posterior instrumented fusion with a good evolution.

## Conclusion

The atlantoaxial subluxation is a potential fatal complication and could be present even after many years of evolution.

## Disclosure of interest

None declared.

